# 

**Published:** 1842-11-12

**Authors:** 


### TO SUBSCRIBERS.

☞ We again beg of our subscribers whose accounts are unsettled to remit us the amount due. We hope that those to whom we have extended credit will feel the justice of this request, and at once discharge their arrears. Our thanks are due to the large number of distant subscribers who have punctually forwarded their subscriptions, and we cannot doubt that those who have not yet done so will acknowledge the present appeal.

Remittances *by mail, at our risk,* in paper current in the states in which subscribers reside, offer the best mode of paying distant subscriptions. Postmasters may legally frank such remittances.

Published EVERY SATURDAY by J. C. TURNPENNY, N. E. corner of Spruce and Tenth streets.

